# Dispersal and adaptation strategies of the high mountain butterfly *Boloria pales* in the Romanian Carpathians

**DOI:** 10.1186/s12983-018-0298-1

**Published:** 2019-01-17

**Authors:** Stefan Ehl, Niklas Böhm, Manuel Wörner, László Rákosy, Thomas Schmitt

**Affiliations:** 1 Biogeography, Trier University, Universitätsring 15, D-54286 Trier, Germany; 2 Senckenberg German Entomological Institute, D-15374 Müncheberg, Germany; 3 FÖA Landschaftsplanung GmbH, D-54296 Trier, Germany; 4 Department of Taxonomy and Ecology, Babeş-Bolyai University, RO-400084 Cluj-Napoca, Romania; 5 Entomology, Department of Zoology, Institute of Biology, Faculty of Natural Sciences I, Martin Luther University Halle-Wittenberg, D-06099 Halle (Saale), Germany

**Keywords:** Mark-release-recapture, *Boloria pales*, Soft protandry, Grazing, Habitat quality, Dispersal behaviour, Conservation, Risk spreading, Nectar sources

## Abstract

**Background:**

Habitat quality is one main trigger for the persistence of butterflies. The effects of the influencing biotic and abiotic factors may be enhanced by the challenging conditions in high-alpine environments. To better our knowledge in this field, we performed a mark-release-recapture study with *Boloria pales* in the Southern Carpathians*.*

**Methods:**

We analysed population structure, movement and foraging behaviour to investigate special adaptations to the alpine environment and to reveal differences between sexes. We compared these aspects in one sector with and one sector without grazing to address the effects of grazing intensity on habitat quality.

**Results:**

We observed “soft” protandry, in which only a small number of males appeared before females, and an extended emergence of individuals over the observed flight period, dividing the population’s age structure into three phases; both observations are considered adaptations to high mountain environments. Although both sexes were mostly sedentary, movement differences between them were obvious. Males flew larger distances than females and were more flight-active. This might explain the dimorphism in foraging behaviour: males preferred nectar sources of Asteraceae, females Caprifoliaceae. Transition from the grazed to the ungrazed sector was only observed for males and not for females, but the population density was higher and the flight distances of the individuals were significantly longer on the grazed sector compared with the ungrazed one.

**Conclusion:**

Soft protandry, an extended emergence of the individuals and an adapted behavioural dimorphism between sexes render to represent a good adaptation of *B. pales* to the harsh environmental conditions of high mountain ecosystems. However, land-use intensity apparently has severe influence on population densities and movement behaviour. To protect *B. pales* and other high-alpine species from the negative consequences of overgrazing, areas without or just light grazing are needed.

**Electronic supplementary material:**

The online version of this article (10.1186/s12983-018-0298-1) contains supplementary material, which is available to authorized users.

## Introduction

The quality of habitats is of great importance for the adaptation of populations and hence is one major driver for the evolution of species [1]. Moreover, persistence of a species in a particular habitat is primarily depending from the ability to adapt to the respective living conditions [2]. However, comparatively little is known about adaptations to environmentally harsh environments. This lack of studies partly results from the complicated study conditions under such hostile environmental circumstances. Consequently, the main focus in autecological research has been on species whose ecological demands and specialisations are linked to conditions in temperate zones. High mountain systems are one important ecosystem type with extreme environmental conditions. These areas exhibit harsh environmental conditions, such as cold snaps that may occur at any time of year [3–5]. Furthermore, climate change and direct human activities aggravate the negative effects of these challenging environments on species’ living conditions. To survive, high mountain species consequently have to be adapted to these unpredictable environmental circumstances [6, 7]. Consequently, habitats like high-alpine areas offer excellent conditions to study more general biogeographical and evolutionary questions.

Specific adaptions of butterflies to harsh climatic conditions have been unravelled in several studies [8–11]. However, little is known about local adaptions and persistence of metapopulations of high-alpine Lepidoptera, especially for species of high-alpine systems other than the European Alps and the North American Rocky Mountains [12–14]. Additionally, the effects of land-use have recently become of increasing interest in studies concerning the impact assessment of land-use changes on the ecology of high-alpine regions and species [15–17]. Thus, Tappeiner and colleagues [18] predicted negative consequences resulting from land-use intensification, especially for species of formerly extensively used alpine habitats. The negative effects of changes in land-use and regional climate have the potential to particularly impact species with limited dispersal, with habitat changes in particular reducing the persistence probabilities of metapopulations [19].

Therefore, we conducted a field study in the Southern Carpathians to investigate general and local adaptations of the shepherd’s fritillary *Boloria pales*, a butterfly species with a disjunct distribution in the alpine belt of several European high mountain systems [20–22]. In general, Romania’s butterfly diversity is linked to the relatively large number of natural and semi-natural habitat types, including a variety of different grassland types, which are a heritage of the Romanian traditional way of living [23]. These grasslands are maintained by extensive grazing, manual mowing and other traditional agricultural techniques, often without the application of agrochemicals [24]. Changes in these traditional agricultural practices have already been identified and predicted as one major threat to Romania’s butterfly diversity [23, 24]. At high altitudes in the Carpathians, which are mostly dominated by natural grasslands, the main threat is the constantly increasing number of livestock, resulting in a continuously increasing rate of overgrazing.

In Romania, *B. pales* is a rare species restricted to some few locations of natural high altitude grasslands in the southern Carpathians [25]. Even at its possibly most important stronghold in the Bucegi Mountains, changes in land-use are apparent, especially changing grazing regimes [26, 27]. We therefore aim to reveal the demographic structure and senescence within one metapopulation of *B. pales* over one entire flight period. We intend to understand whether the species’ regional adaptation to these high mountain habitats is sufficient to maintain a protandrous population structure with all its advantages [28–30] or whether a close synchronization of the emergence of both sexes is necessary for surviving the high mountain challenge, as for example in the case of *Euphydryas aurinia glaciegenita* [31, 32]. We further analyse whether emergence is restricted to a short time window as in many univoltine lowland butterfly species [33–38], or is extended over a longer period to spread the risk of encountering highly unfavourable conditions caused by the unpredictable weather in high mountain ecosystems. The latter response is known for populations of this [39] and other species [40] assayed in the Alps.

While male butterflies are often assumed to be the more flight-active sex, for example due to patrolling for females [35, 37, 41–43], we test for differences in dispersal behaviour and movement patterns between sexes, by analysing the distances moved and the mean recruitment of flying individuals in the population for both sexes. It is already known that some male and female butterflies prefer nectar containing different ingredients [44, 45]. For example, males of *Polyommatus bellargus* prefer high amounts of sucrose and total sugar, while females opt for glucose and amino-acids [46]. Therefore, we test whether each sex exhibits preferences for different nectar sources and which flower traits may affect their nectar foraging strategy. Finally, we analyse whether the individuals are specialised on specific flowers or are mostly unspecific, as postulated for *B. pales* as a species [47].

Independent from the sex-dependent needs, habitat quality in general has an important impact on the behaviour and movement patterns of animals [48]. However, habitat quality for many butterfly species is strongly influenced by grazing intensity and general management. As strong changes in the use of high-altitude grasslands are apparent in many high mountain areas, we examine the effects of grazing intensity on behavioural traits and movement patterns of *B. pales* by studying patches within a metapopulation with different degrees of disturbance by sheep and cow pasturing. In this context, we also analyse the differential response of both sexes to habitat quality in combination with population density.

To tackle these general aspects, we examined the population ecology of *B. pales* in the Southern Carpathians from 02 July to 02 September 2014, thus covering most of the flight period, using mark-release-recapture. We selected one of the largest metapopulations known in Romania, in the Bucegi Mountains, a plateau-like region traditionally used as high-altitude sheep pasture, where grazing pressure has strongly increased during the past few decades. These data in combination with results obtained from the eastern Alps [39] allow the analysis of the adaptation of this species to high mountain conditions, the assessment of the different behavioural traits of both sexes and a better understanding of their needs, as well as deepening our understanding of the consequences of the increasing human impact in some parts of the European high mountain regions. We conclude with some general conclusions for nature conservation in high mountain ecosystems.

## Materials and methods

### Study species

The alpine butterfly *Boloria pales* (Denis & Schiffermüller, 1775) belongs to the family Nymphalidae. Except for the northern European mountains (Scandes, Scottish Highlands), the species occurs in most high mountain systems of Europe from 1500 to 2700 (maximum 3100) m asl.; in the Pyrenees, populations are known as low as 1100 m asl. [20–22, 49]. The species lives at flower-rich high-altitude grasslands, where adults feed at blossoms of the respectively dominant flowering plants [20]. The larvae feed on *Viola, Plantago* or *Polygonum* species [21, 49–51]. *B. pales* usually occurs in one generation per year [20, 21, 49, 50] and the flight period is comparatively long, often more than eight weeks; mostly from late June or early July to early September [21, 49, 51]. The larvae hibernate as caterpillars (of the 1st instar) and grow after the snow has melted in springtime [20]. Due to the often high abundance at their flight places, the species is a suitable model organism for analysing the population dynamics of high mountain species.

### Study area

**Fig. 1:**
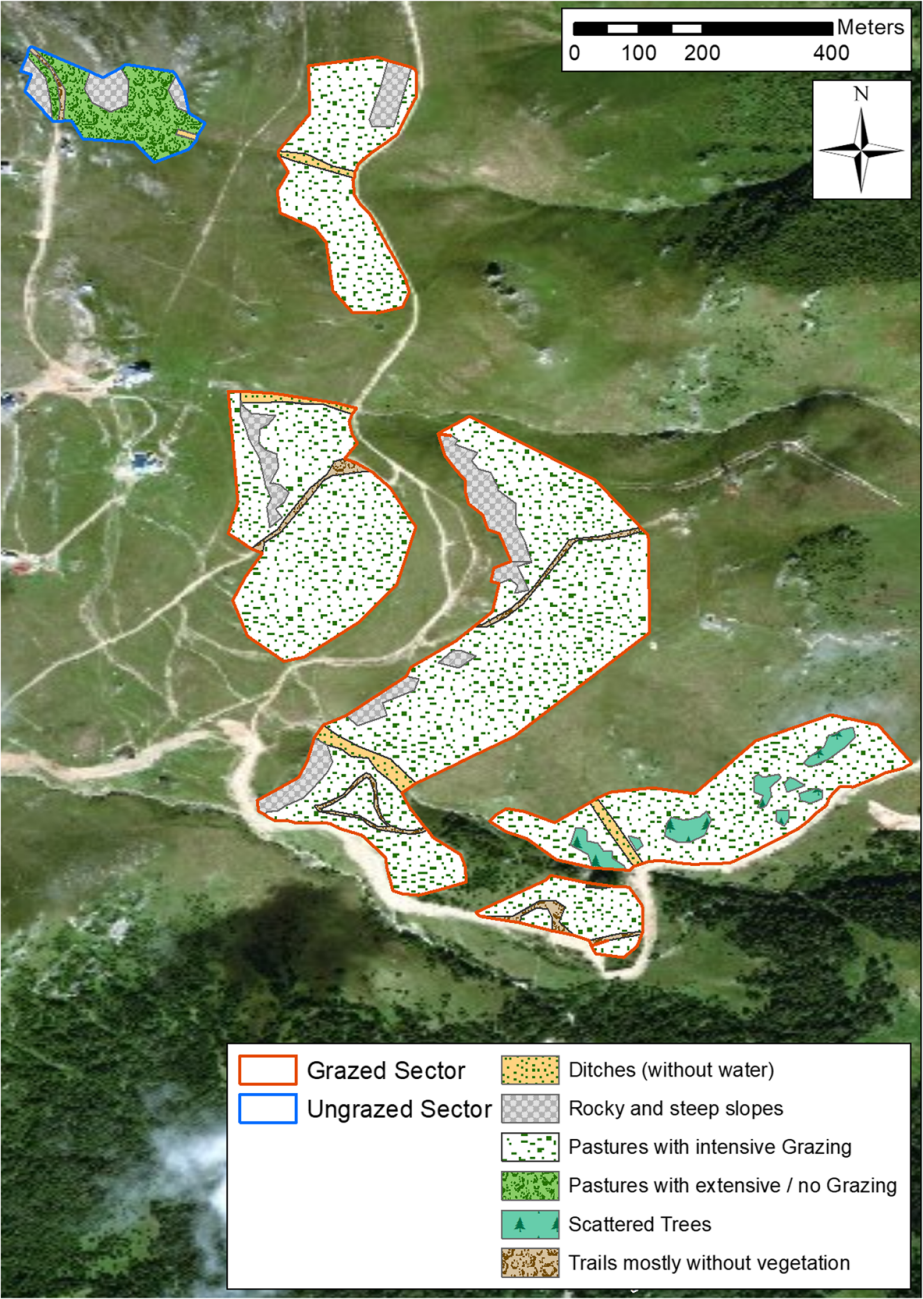
Map showing the study area in the Bucegi Natural Park with sector boundaries and habitat types

The mark-release-recapture (MRR) study area was located in the Bucegi Natural Park / Parcul Natural Bucegi (N 45°21′, E 25°30′) near the hiking trail Cota 2000 in the Southern Carpathians (Sinaia, Prahova county, Romania). The area studied had no natural boundaries and a mostly homogeneous vegetation cover. It was divided into two sectors because of the different grazing intensity (distance between sectors ca. 150 m; altitudinal difference: ca. 30 m). The lower sector of 14.2 ha reached from 1660 to 1980 m asl., the upper sector of 2.2 ha reached from 2010 to 2090 m asl. Both sectors were characterized by alpine and subalpine grasslands (partly dominated by *Carex curvula*) with generally little inclination, but interspersed with steep, partly rocky slopes and some few cliff edges (Fig. 1). At the lower sector, grazing by a herd of cows (about 80 individuals) and two herds of sheep (about 200 and 350 individuals) was observed each sampling day, whereas the upper sector was not used as pasture, probably because of the proximity to the Cota 2000 trail and other nearby hiking trails. Consequently, although similar in their floristic composition, the abundance of flower heads was considerably higher in the upper sector if compared with the lower areas. In the following, we call the lower sector grazed sector and the upper sector ungrazed sector.

### Mark-release-recapture (MRR)

We performed a MRR study from 02 July to 02 September 2014, i.e. almost the entire flight period of *B. pales*. Weather conditions during our time of study were not unusual for the Bucegi Mountains. However, days with rainfall were frequent (21 of 63 days) with only two longer periods without precipitation, i.e. 31 July to 7 August and 25 August to 2 September. On the other hand, rainy periods were never longer than three continuous days. The highest temperature was reached at 14 August with about 20 °C, whereas the coldest temperature was recorded in the night 28 to 29 August with about − 1 °C.

On each day with suitable weather conditions (weak or moderate wind; no rain or snow), we sampled the entire study area, netting all available individuals. On every sampling day, we started at a different point in the area, to ensure that sufficient information was collected from all parts of the study area for every time of the day. The grazed sector was examined over the total study period, whereas the ungrazed sector was only studied from 13 August to 02 September. The great initial expenditure of time to examine the grazed sector did not permit the analysis of the additional ungrazed sector before.

Every captured butterfly was marked with a durable fineliner using an individual code consisting of a letter for the sampling day and a running number, always starting with one every day. Prior to release, we recorded the following information in addition to the individual code: sex, GPS position of capture point (with a maximal deviation of three metres), capture time, wing wear (scored from 1 for fresh to 4 for heavily damaged [42, 52, 53]), behaviour (i.e. flying, resting, feeding, interaction) and, if applicable, the nectar plant. For all recaptures, made at least one day after the last capture event to avoid capture-release trauma [54, 55], we recorded identical information. With the information on wing conditions, we classified the age of the butterfly individuals [54, 56, 57]. Based on all captured individuals and all capture events, we calculated the age structure along the observed flight period (values based on less than five individuals per sex and day were excluded). An analysis correlating the time elapsed from capture to the next recapture with the average wing decay over this time period allowed the assessment of the sex-dependent wing decay rate per time unit. With the four behavioural categories, we performed χ^2^ homogeneity tests to detect differences in the behaviour between sexes (to avoid the bias of individual preferences, the behaviour of each individual was included only once at its first capture event).

Furthermore, we performed five plant inventories with an adjusted method following the Braun-Blanquet system [58] to assess plant species and their abundance [59]. We also estimated the cover of all plant species with the Braun-Blanquet scale, covering the entire study area and not just delimited areas. With these data, we calculated the proportion of every plant species in relation to the total plant cover during the study period. Assuming a non-random use of nectar sources by the butterflies, we compared the observed use of nectar sources with the one expected if flower selection was at random using χ^2^ tests. This test was performed separately for both sexes. With the Jacobs’ index of selection [60] (Jacobs interpretational classification of Jacobs` index for our study: 1 to 0.33 preference, 0.33 to − 0.33 neutrality, − 0.33 to − 1 avoidance) and Bailey’s confidence intervals at *p* values < 0.05 [61, 62], we tested for differences in preferred nectar sources at the plant families and plant genera level. All univariate statistics were calculated in SPSS 22.0 [63].

### Population demography

In the program package MARK 8.0, we used the module POPAN 5.0 [64] to estimate daily population sizes based on the Jolly-Seber model (for open populations) [65]. We estimated population sizes separately for the sexes and sectors. POPAN 5.0 estimates three primary parameters: survival probability (*phi*), capture probability (*p*) and proportional recruitment (*pent*). These parameters may be constant (.), dependent on sex (*g*), respond to time in factorial (*t*), linear (*T*) or quadratic (*T*^*2*^) manner, or display additive (*g* + *t*, *g* + *T, g* + *T*^*2*^) or interactive (*g* x *t*, *g* x *T, g* x *T*^*2*^) effects [33, 66, 67]. The capture probability might also depend on the sampling effort (*hours*) [64]. Sampling effort was calculated as the product of time spent conducting field work (ranging from one to six hours per day) and the number of persons involved (ranging from two to four) (see Additional file 1: Table S1). With a second model in the program MARK (Multi-state Recaptures only [64]), we calculated the transition between both sectors *(psi)* in the period from 13 August to 02 September [36, 68–71]. Beside transition between sectors, calculations for survival (*S*) and capture probability (*p*) within both sectors were performed separately for both sexes with this module. As in the first module, these parameters may be constant (*.*), different among sectors (*o*), time (*t*) or linear (*T*) or quadratic (*T*^*2*^) time and can also occur as additive (*o* + *t*, *o* + *T, o* + *T*^*2*^) or interactive (*o* x *t*, *o* x *T, o* x *T*^*2*^) effects. The capture probability might also depend on the sector size (*size*) [64]. After separate Goodness-of-Fit tests for both modules (option: RELEASE), we analysed different combinations of the parameters mentioned above and selected separately for each module the model with the lowest value for the corrected Akaike Information Criterion (AIC_C_) [72, 73] and the smallest numbers of parameters [74] as best supported. With the computed values for Net Birth rates and for rates of proportional recruitment (*pent*), we completed our classification of the age of the butterflies, derived from wing conditions. With the estimated survival probability (*phi*), we computed longevity using the formula of Cook [75]: Longevity = − 1 / log_e_ (*phi*). Additionally, we performed Mann-Whitney U tests to examine significant differences in *phi* between sexes (both sectors pooled and each sector separately), sectors and days with fine (i.e. sun or loose cloudiness) against days with weather conditions unsuitable for butterfly activity (i.e. with a maximal day temperature below 5 °C or with rain or snow events). Further Mann-Whitney U tests were used to examine differences in the transition between both sectors (*psi* from the chosen models). This analysis was performed for both sexes pooled and separately. For tests between sectors, we only used data collected after 13 August, when both sectors were analysed simultaneously. We applied Bonferroni correction for all Mann-Whitney U tests because of the increasing risk of type I errors when making multiple statistical tests based on the same data set.

**Table 1:** Comparison of the best models of the POPAN 5.0 analyses for estimating the daily population sizes of *B. pales* in 2014 in Parcul Natural Bucegi, Romania: Akaike information criterion (AIC_C_) and number of considered parameters

	Model	AICc	Parameters
Grazed sector	*{phi(g x T* ^*2*^ *) p(g + hours) pent(g + t) N(g x t)}*	1712.88	30
	{*phi*(*g* x *T*) *p*(*g* + *hours*) *pent*(*g* + *t*) *N*(*g* x *t*)}	1714.85	32
	{*phi*(*g* x *T*) *p*(*g* x *hours*) *pent*(*g* + *t*) *N*(*g* x *t*)}	1716.26	33
Ungrazed sector	*{phi(g + t) p(g x hours) pent(g + t) N(g x t)}*	1571.84	25
	*{phi(g + t) p(g + hours) pent(g + t) N(g x t)}*	1572.29	23
	{*phi*(*g* + *t*) *p*(*g* + *hours*) *pent*(*g* x *t*) *N*(*g* x *t*)}	1573.97	32

Basic variables: survival rate (*phi*), capture probability (*p*), proportional recruitment (*pent*), total number of individuals (*N*). Dependent variables: sex (*g*); factorial (*t*), linear (*T*) and quadratic (*T*^*2*^) dependency on time. The model with the lowest value for AIC_C_ and the smallest numbers of parameters was chosen as best supported (underlined); for the grazed sector, it was the first model, for the ungrazed sector it was the second model

### Mobility and movement patterns

The GPS data set of capture/recapture events obtained in this MRR study was used to analyse the movement behaviour of *B. pales*. With ArcGIS 10.2.1 (ESRI), we measured the direct distance travelled from the first capture event to the second capture event for all individuals. Based on these data, we tested for differences in mobility between sexes with a Mann-Whitney U test (with Bonferroni-correction). With these single distances, we calculated the inverse cumulative proportion of individuals moving in certain distance classes, with each class representing an interval of 20 m. We fitted these data against two different mathematical models, which are commonly applied to find the best prediction of long-distance movements [31, 33, 37, 42, 76, 77]: the negative exponential function (NEF) and the inverse power function (IPF). To exclude artefacts based on the 20 m interval size, we performed similar analyses with 30 m and 50 m intervals. All calculations were performed separately for both sexes. For the NEF, the relative proportion of individuals moving to distance *D* is.

*I*_*NEF*_ *= a*e^*-kD*^ respective ln *I =* ln *a - kD*.

The parameter *a* represents a scaling constant while *k* is the dispersal constant describing the shape of the exponential curve. Under the IPF, the proportion *I* is expressed as.

*I*_*IPF*_ *= cD*^*-n*^ respective ln *I =* ln *c - n (*ln *D),*

where *c* is a scaling constant and *n* a variable describing the effect of the distance on dispersal [33, 76]. With the calculated coefficient of determination *R*^*2*^ and adjusted coefficient of determination *R*_*adj*_^*2*^ for the computed curves, we chose the best model and interval size to predict the proportion of individuals moving greater distances than those covered by our MRR study [31, 33, 42, 76].

## Results

During 36 field days, we marked 633 individuals (males: 401; females: 232) with a total of 1202 capture events on both sectors (Additional file 2). The recapture ratio was 51.4% for males and40.1% for females. In total, more than half of the capture events were at the ungrazed sector (622, i.e. 51.7%) during the 11 sampling days there. We obtained multiple recapture events (up to six recaptures for both sexes). The longest residence time between capture and last recapture was 24 days for males and 17 days for females, whereas the calculated longevity was 4.5 ± 0.5 SD days for males and 3.3 ± 0.6 SD days for females.

For comparing the estimated survival probabilities (*phi*), we used Mann-Whitney U tests because none of the data was normally distributed and all samples from the POPAN calculation were independent from each other. The comparison of the sexes pooled for both sectors revealed significant differences (critical *p* value with Bonferroni correction: 0.05 / 4 = 0.0125) between the daily survival rates of males and females (males: 0.80 ± 0.14 SD; females: 0.74 ± 0.17 SD; U test: *p* = 0.002). These significant differences were also found on the grazed sector (males: 0.80 ± 0.14 SD; females: 0.73 ± 0.16 SD; U test: *p* < 0.001), but not on the ungrazed sector (males: 0.81 ± 0.15 SD; females: 0.77 ± 0.19 SD; U test: *p* = 0.649). No significant differences between the sectors were found, neither for males (U test: *p* = 0.893) nor females (U test: *p* = 0.704). Significantly higher survival probabilities during days with suitable against days with harsh weather conditions were observed for both sectors and sexes (U tests: grazed sector: 0.85 ± 0.03 vs. 0.59 ± 0.17, *p* < 0.001; ungrazed sector: 0.86 ± 0.13 vs. 0.61 ± 0.11, *p* = 0.001; males: 0.88 ± 0.06 vs. 0.65 ± 0.16, *p* < 0.001; females: 0.83 ± 0.07 vs. 0.55 ± 0.15, *p* < 0.001; all values are means ± SD).

### Demography – Grazed sector

**Fig. 2:**
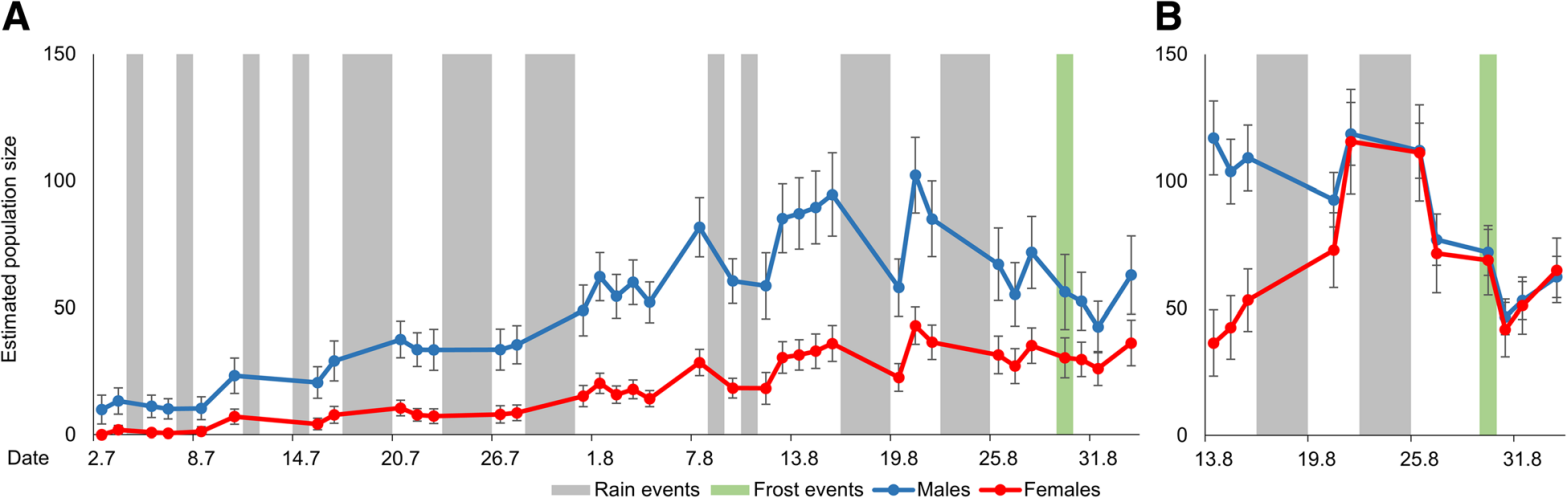
Estimated population size of *B. pales* for every sampling day. **a** Grazed Sector, (**b**) Ungrazed Sector; Vertical bars represent days with harsh weather conditions (grey bars: no sampling on days with rain events, green bar: day after frost event at night); error bars represent the standard error of the calculated population size from the program MARK; note that the graphs have different units on the x-axis

The most plausible model estimated a total population size of 550 males (± 45 SE) and 242 females (± 25 SE), i.e. a population density of 39 males (± 3 SE) and 17 females (± 2 SE) per hectare (Table 1). The estimated gender ratio was 2.3 males per female. The phenology based on the POPAN model (Fig. 1a) was protandrous. Thus, the number of males started to increase from the beginning of our sampling, whereas females only noticeably increased some three to four weeks later. With the beginning of the females’ emergence, the phenologies of both sexes ran more or less in parallel. Both sexes increased slowly until late July, then rose more quickly to a peak at 20 August. Thereafter, the male population decreased, whereas the female population remained at a mostly constant level. At the very end of the study period, both sexes showed a slight increase. The sensitivity to bad weather conditions (rain or wind events) was moderate for both sexes at the beginning of the study. In August, when strong winds occurred in combination with strong rainfall events, the effects of weather were greater, but more obvious in males than females (Fig. 2a).

### Demography – Ungrazed sector

The most plausible model with the second lowest value for the AIC_C_ and the smallest numbers of parameters estimated 271 males (± 13 SE) and 270 females (± 31 SE), i.e. 123 males (± 6 SE) and 123 females (± 14 SE) per hectare (Table 1). The estimated gender ratio was practically equal (one male per female). Although the ungrazed sector was studied for a shorter period of time, the estimated population sizes of both sectors revealed a by far higher density on the ungrazed sector. The ungrazed sector’s phenology had a higher male population size until 20 August, when both sexes reached their peak at a nearly identical population size. After this peak, the female population stayed constant until 25 August and exceeded the male population (which declined slightly) for the rest of the study period. Thereafter, both sexes decreased in parallel and slightly increased again at the end of August (Fig. 2b). The sensitivity to bad weather conditions was similar to the results on the grazed sector.

### Wing conditions

The deterioration rate of wing condition was assessed by comparing the condition of captured and first recaptured individuals. For both sectors combined and each sector separately, both sexes showed a strong correlation between wing decay and the time elapsed between capture events (Pearson correlations, all *p* < 0.001). The average time needed for the deterioration of wing condition by one category was shorter for males than for females (males: 13.5 days ±1.3 SE; females: 15.6 ± 2.4 SE).

Wing conditions of *B. pales* were good to very good for most of the individuals throughout the observed flight period. Even at the end of the study period (after mid-August), the wings of most captured individuals were in good condition (i.e. about 75% category 1). Seriously damaged wings (i.e. categories 3 and 4) were exceptions throughout, and freshly emerged individuals (verified by the excretion of a red liquid from the abdomen) of both sexes were always recorded, even at the end of the study period. Thus, old butterflies must have been constantly replaced by freshly emerged ones until mid-August or even later in the flight period.

**Fig. 3:**
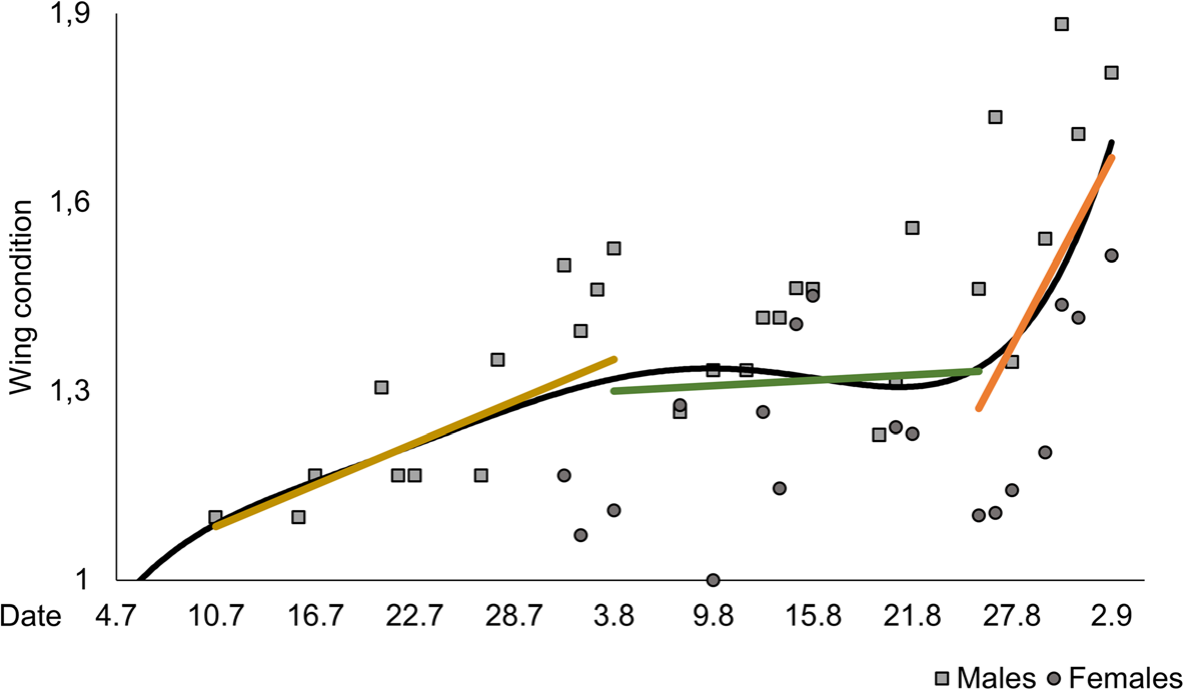
Age structure calculated based on the wing conditions of *B. pales*. Days with less than five data were excluded (no analysed data prior to 10 July); black trend line for weighted mean value of the total population (fourth-degree polynomic function); coloured linear trend lines for the three phases of the aging process (yellow: 10 July–03 August, green: 03 August–25 August, orange: 25 August–02 September). Only data of wing decay from the first to the second capture event of each individual were used for the calculations

At the beginning of the studied flight period, average wing condition deteriorated gradually until 03 August (Pearson Correlation: *p* = 0.004, *R*^2^ = 0.59, *R*_*adj*_^*2*^ = 0,54). Afterwards, this remained at the same average level until 25August (Pearson Correlation: *p* = 0.734, *R*^2^ = 0.01, *R*_*adj*_^*2*^ = 0,00), when the start of a second phase of average wing deterioration was observed (Pearson Correlation: *p* = 0.021, *R*^2^ = 0.69, *R*_*adj*_^*2*^ = 0,63) (Fig. 3). This tripartition was also supported by a fourth-degree polynomic function obtaining the best adjusted coefficient of determination (*R*_*adj*_^2^ = 0.60) and coefficient of determination (*R*^*2*^ = 0.66) (Additonal file 2: Table S2).

**Fig. 4:**
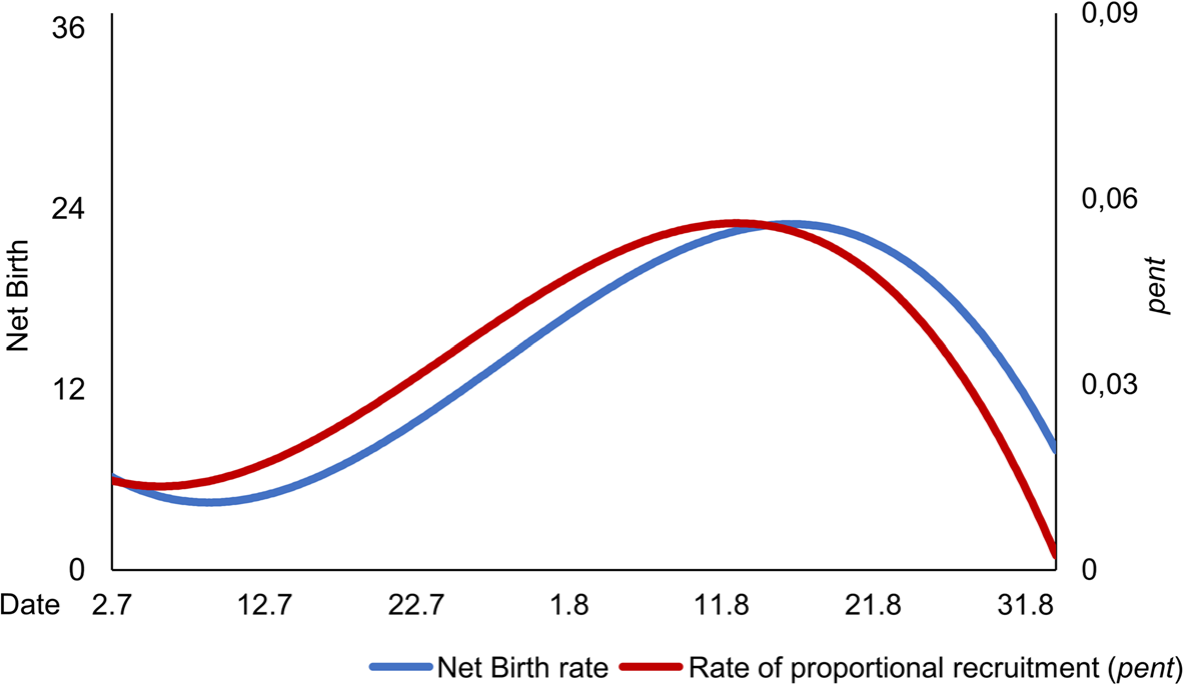
Net Birth rate and rate of proportional recruitment (*pent*) and of *B. pales* calculated with POPAN 5.0 in the program MARK. Red trend line for weighted mean value of *pent* (third-degree polynomic function); blue trend line for weighted mean value of Net Birth (third-degree polynomic function)

Hence, deterioration in wing conditions over the observed flight period can be divided into three different phases (Fig. 3), which are well supported by the development of the Net Birth rates and the rates of proportional recruitment (*pent*) calculated with (Fig. 4; third degree polynomic function obtaining the best adjusted coefficient of determination (*R*_*adj*_^2^) for both values); both rates show long initial increases with maxima around 15 August and rapid decrease thereafter:

- Initial phase: Slight deterioration of average wing condition. Most individuals are still young and their mortality rate is comparatively low. Therefore, the replacement of the few older individuals by newly emerging individuals cannot compensate the overall ageing of the already existing individuals. (emergence > mortality).
- Equilibrium phase: Dying older individuals are directly replaced by the large number of newly emerging individuals, which completely compensate the ageing process of the existing individuals. As a consequence, the population has a mostly constant age structure (emergence = mortality).
- Ageing phase: Due to the small number of newly emerging individuals, the ageing process of the population is not compensated. The high mortality rate of older individuals is not sufficient to counteract this ageing process (mortality > > emergence).

Equation, coefficient of determination (*R*^*2*^) and adjusted coefficient of determination (*R*_*adj*_^*2*^) for trend lines of the fourth-degree polynomic function:

y = 8E-07 × ^4^–0.14 × ^3^ + 8725.23 × ^2^ - 2E+ 08x + 3E+ 12; *R*^2^ = 0.66; *R*_adj_^2^ = 0.60.

Yellow: *R*^2^ = 0.59; *R*_*adj*_^*2*^ = 0,54; *p* = 0.004.

Green: *R*^2^ = 0.01; *R*_*adj*_^*2*^ = 0,00; *p* = 0.734.

Orange: *R*^2^ = 0.69; *R*_*adj*_^*2*^ = 0,63; *p* = 0.021.

Equations, adjusted coefficients of determination (*R*_*adj*_^*2*^) for trend lines of the third-degree polynomic function:

Net Birth rate: y = 0.0007 × ^3^ + 84.57 × ^2^ - 4E+06x + 5E+ 10; *R*_adj_^2^ = 0.29.

Rate of proportional recruitment (*pent*): y = −2E-06 × ^3^ + 0.2005 × ^2^–8534.2x + 1E+ 08; *R*_adj_^2^ = 0.35.

### Behaviour and foraging

Significant behavioural difference existed between sexes. Thus, females fed significantly more often than males, whereas males flew significantly more often than females; a similarly portion of both sexes was resting when sampled (χ^2^-homogeneity test: χ^2^ = 16.60, *df* = 3, *p* = 0.001). A significant behavioural difference between sexes was also observed when only the grazed sector was considered (χ^2^ = 12.18, *df* = 3, *p* = 0.007), but not for the ungrazed sector alone (χ^2^ = 3.86, *df* = 3, *p* = 0.277). In general, the individuals are more often flying on the grazed than on the ungrazed sector, whereas the individuals on the ungrazed sector were more frequently resting (males) or feeding (females) (χ^2^ = 44.30, *df* = 3, *p* < 0.001). This difference was also significant for each sex separately (males: χ^2^ = 33.11, *df* = 3, *p* < 0.001; females: χ^2^ = 10.54, *df* = 3, *p* = 0.014) (Table 2).

**Table 2:** Percentage of individuals of *B. pales* in four different behavioural categories

Category	Males total	Females total	Males grazed	Males ungrazed	Females grazed	Females ungrazed
Flying	44.5%	27.4%	53.6%	32.9%	34.8%	20.5%
Resting	29.0%	33.9%	23.6%	35.8%	33.9%	33.9%
Feeding	23.7%	37.8%	19.6%	29.0%	29.5%	45.6%
Interaction	2.8%	1.0%	3.2%	2.3%	1.8%	0.0%

x

Butterflies fed only on plants belonging to the families Asteraceae and Caprifoliaceae, with a clear preference of males for Asteraceae (84 of 93 visits) and of females for Caprifoliaceae (61 of 86 visits). The predominant nectar source was *Hieracium aurantiacum* for males (73 of 93 visits) and *Scabiosa lucida* for females (62 of 88 visits). This sex-related difference in foraging behaviour was significant (χ^2^-homogeneity test: plant families χ^2^ = 72.48, *df* = 3, *p* < 0.001; genera χ^2^ = 84.04, *df* = 4, *p* < 0.001).

Comparing the observed and expected (from plant social inventories and the assumption of random flower-use) nectar sources revealed significant differences for both sexes (males: χ^2^ = 297.20, df = 33, *p* < 0.001; females: χ^2^ = 1171.14, df = 33, p < 0.001). Males preferred the genera *Hieracium* (73 of 93 visits) in the Asteraceae, whereas the genera *Scabiosa* and *Leontodon* were used as expected (cf. Jacobs` index and Bailey’s confidence intervals in Table 3). For females, preference existed for the genus *Scabiosa,* whereas there were no preferences or even avoidance for all genera of the Asteraceae observed during our study (cf. Jacobs` index and Bailey’s confidence intervals in Table 3).

**Table 3:** Results for the selection of nectar sources of *Boloria pales* using Jacobs’ index of selection (Jacobs 1974; interpretational classification of Jacobs` index for our study: 1 to 0.33 preference, 0.33 to − 0.33 neutrality, − 0.33 to −1 avoidance) and Bailey’s confidence intervals at *p value* < 0.05 (Bailey, 1980; Cherry, 1996); rating: “+” preference nectar source, “=” neutrality, “-”avoidance

	Category	Observed visits	Proportion expected	Proportion used	Jacobs` index	Jacobs` index rating	Bailey’s confidence intervals	Bailey’s confidence intervals rating
Plant family								
Males	Asteraceae	84	0.208	0.903	0.945	+	(0.744;0.969)	+
	Caprifoliaceae	9	0.035	0.097	0.495	+	(0.020;0.226)	=
Females	Asteraceae	25	0.208	0.284	0.204	=	(0.139;0.460)	=
	Caprifoliaceae	61	0.035	0.693	0.968	+	(0.514;0.843)	+
Plant genera								
Males	*Hieracium* (Asteraceae)	73	0.245	0.785	0.837	+	(0.604;0.895)	+
	*Leontodon* (Asteraceae)	11	0.112	0.118	0.029	=	(0.030;0.254)	=
	*Scabiosa* (Caprifoliaceae)	9	0.106	0.097	−0.049	=	(0.020;0.226)	=
Females	*Carduus* (Asteraceae)	2	0.081	0.023	−0.578	–	(0.001;0.122)	=
	*Carlina* (Asteraceae)	1	0.062	0.012	−0.690	–	(0.005;0.099)	=
	*Hieracium* (Asteraceae)	14	0.245	0.163	−0.250	=	(0.052;0.316)	=
	*Leontodon* (Asteraceae)	8	0.112	0.093	−0.103	=	(0.016;0.227)	=
	*Scabiosa* (Caprifoliaceae)	61	0.106	0.709	0.907	+	(0.514;0.843)	+

### Mobility and movement patterns

**Fig. 5:**
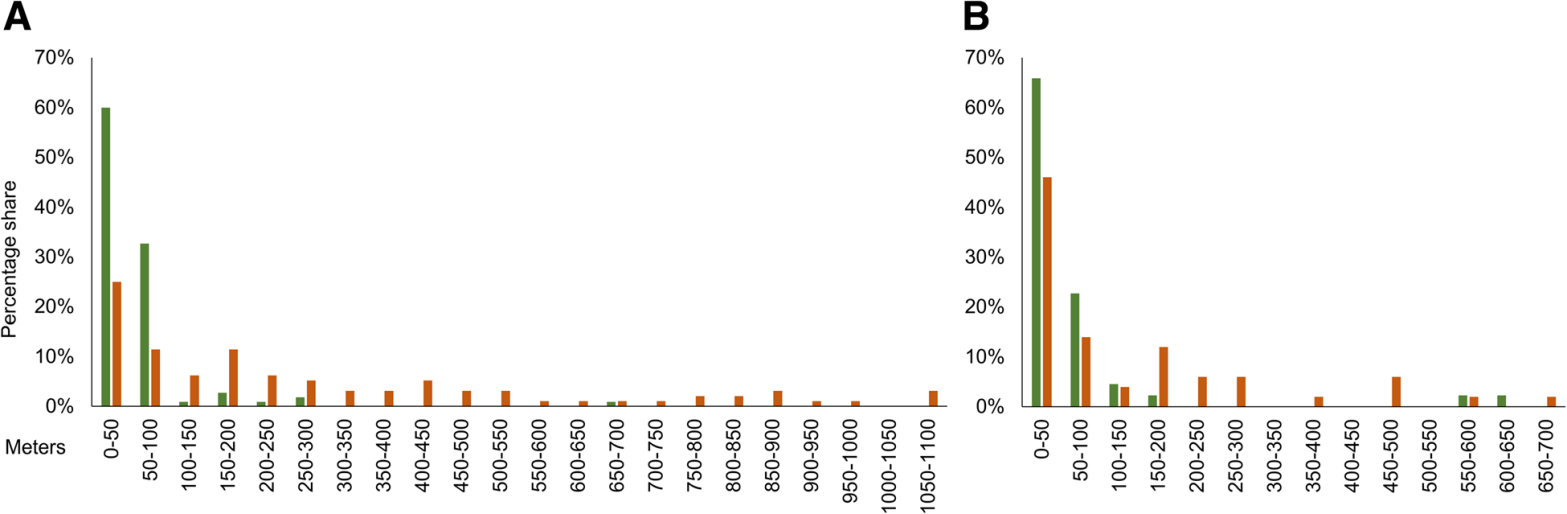
Percentage of recaptured individuals of *B. pales* in combination with their movement distances between capture and first recapture event. The distances are divided into 50 m intervals; (**a**) males*,* (**b**) females. Green bars represent the ungrazed sector, orange bars represent the grazed sector

Most of the *B. pales* individuals were sedentary. Especially on the ungrazed sector, 93% of the males and 69% of the females did not move more than 100 m. Although both sexes flew larger distances on the grazed sector, most of the females were also sedentary and did not fly more than 200 m (i.e. 76%). In contrast, males at the grazed sector were more flight-active, a larger percentage moved distances exceeding 200 m (i.e. 46%), and even movements of more than 700 m were frequently observed (Fig. 5a and b). The longest observed distance moved was 1096 m for males and 654 m for females. Both distances were observed starting at the grazed sector, whereby also large distances were observed when individuals started on the ungrazed sector (males: 810 m from the first to the second recapture event; females: 615 m from the first capture to the first recapture event). Hence, long movement distances were observed for both sexes on both sectors.

Consequently, the mean distance moved by males (288 m ± 30 SD) was significantly longer than for females (138 m ± 22 SD) on the grazed sector (U test: *p* = 0.002). On the ungrazed sector, no significant difference between males (58 m ± 8 SD) and females (73 m ± 18 SD) was observed (U test: *p* = 0.914). Nevertheless, in contrast to the grazed sector, the value for females was higher than for males. Analysing the sexes separately, the mean distance moved was significantly longer on the grazed than on the ungrazed sector for males (U tests: *p* < 0.001), but not for females (U test: *p* = 0.045). The critical *p* value with Bonferroni correction was 0.05 / 2 = 0.025).

The highest stability indices (i.e. *R*_*adj*_^*2*^) of fitting the inverse cumulative proportion values of individuals moving certain distance classes to NEF and IPF were obtained for 50 m intervals, for both sexes and both sectors (exception: NEF for females on the grazed sector; males have similar values for all three intervals on the ungrazed sector). On the grazed sector, NEF had higher *R*_*adj*_^*2*^ values than IPF in all cases, whereas IPF always had higher *R*_*adj*_^*2*^ values on the ungrazed sector (Table 4). Based on both algorithms and 50 m intervals, we estimated the proportion of individuals moving distances of 1 km, 2 km, 3 km, 5 km and 10 km (Table 5).

**Table 4:** Adjusted Stability Index (*R*_*adj*_^*2*^) for IPF and NEF based on 20 m, 30 m and 50 m intervals calculated with movement distances of *B. pales*

		20 m intervals		30 m intervals		50 intervals	
		IPF	NEF	IPF	NEF	IPF	NEF
Grazed sector	Males	0.77	0.97	0.78	0.97	0.82	0.98
	Females	0.86	0.98	0.84	0.97	0.87	0.97
Ungrazed sector	Males	0.94	0.71	0.94	0.71	0.94	0.71
	Females	0.85	0.54	0.85	0.56	0.86	0.59

Underlined numbers represent the highest values for the adjusted Coefficient of determination (*R*_*adj*_^*2*^)

**Table 5:** Percentage share of individuals of *B. pales* which would travel 1 km, 2 km, 3 km, 5 km or 10 km; calculated with the NEF and IPF based on 50 m intervals

	Distance	Intervall Number	IPF Males	NEF Males	IPF Females	NEF Females
Grazed sector	1 km	20	7.66	4.98	2.23	0.37
	2 km	40	3.54	0.23	0.81	1.26^−3^
	3 km	60	2.25	0.01	0.45	4.29^−6^
	5 km	100	1.27	2.22^−5^	0.21	4.99^−11^
	10 km	200	0.59	4.56^−12^	0.08	2.32^−23^
Ungrazed sector	1 km	20	0.26	0.05	1.47	0.48
	2 km	40	0.07	8.36^−5^	0.62	7.63^−3^
	3 km	60	0.03	1.42^−7^	0.37	1.21^−4^
	5 km	100	0.01	4.07^− 13^	0.20	3.08^−8^
	10 km	200	2.91^−3^	5.70^−27^	0.08	3.15^−17^

For comparing the estimated transition probabilities (*psi*), we used Mann-Whitney U tests because none of the data was normally distributed and all samples from the calculations of the most plausible model of the Multi-state Recaptures (males second model, females first model; Table 6) were independent from each other. Significant differences (critical *p* value with Bonferroni correction: 0.05 / 4 = 0.0125) between sexes were observed in all cases (U test: transition ungrazed to grazed sector: males 0.060 ± 0.020 vs. females 0.012 ± 0.008, *p* < 0.001; transition grazed to ungrazed: males 0.100 ± 0.020 vs females 0.029 ± 0.027, *p* = 0.008; complete transition: males 0.079 ± 0.030 vs. females 0.020 ± 0.017, *p* < 0.001). The transition probabilities between sectors were not significantly different for females (U tests: *p* = 0.737) and for both sexes combined (U test: transition ungrazed to grazed sector 0.035 ± 0.021 vs. transition grazed to ungrazed sector 0.065 ± 0.060, *p* = 0.068). However, the transition probabilities between sectors was significantly different for males (U test: *p* < 0.001; all values are means ± SD) with more males taking the direction to the ungrazed sector than the reverse.

**Table 6:** Comparison of the best models of the Multi-state Recaptures; only analyses for estimating the daily population sizes of *B. pales* in 2014 in Parcul Natural Bucegi, Romania: Akaike information criterion (AIC_C_) and number of considered parameters

	Model	AICc	Parameters
Males	{*S*(*o* x *t*) *p*(*size*) *psi*(*o* + t)}	1525.11	22
	*{S(o x t) p(size) psi(o + T* ^2^ )}	1525.13	17
	{*S*(*o* x *t*) *p*(*size*) *psi*(*o* + *T*)}	1525.62	17
Females	*{S(t) p(.) psi(o x t)}*	617.84	11
	{*S*(*o* + *t*) *p*(*.*) *psi*(*o* x *t*)}	619.79	12
	{*S*(*t*) *p*(*o*) *psi*(*o* x *t*)}	621.87	13

Basic variables: survival (*S*), capture probability (*p*), transition between states respectively both sectors (*psi*). Dependent variables may be: constant (.), different among sectors (*o*), factorial (*t*), linear (*T*) and quadratic (*T*^*2*^) dependency on time. Capture probability may also depend on sector size (*size*). The model with the lowest value for AIC_C_ and the smallest numbers of parameters was chosen as best supported (underlined); for the males it was the second model, for the females it was the first model

## Discussion

### Adaptations to high mountain ecosystems

The time-shifted appearance of males and females within a species [6, 54] is a common phenomenon in many Lepidoptera species, e.g. *Limenitis camilla* [78], *Bupalus piniarius* [79], *Gonepteryx rhamni* [80], *Euphydryas desfontainii* [81]*.* There are different observations about the consequences of protandry for butterfly population. On the one hand, disadvantages for butterflies are known, i.e. a temporal mismatch between males and females [82, 83], but most studies support the benefits for males [30] and females [29, 84]. Whether protandry is beneficial or not might strongly depend on the degree of adaptation to the local environment. Hence, its existence in a butterfly population is assumed to be an indicator for its adaptation to the prevailing (climatic) conditions and resources. For example, studies with different populations of *Euphydryas aurinia* demonstrated that well-adapted lowland populations of the species showed a protandrous phenology [33, 53, 84, 85], whereas a population at high altitudes in the eastern Alps did not [31]; this non-protandrous population most likely represented the upper edge of the species’ ecological niche.

In our study, protandry was observed in the surveyed Southern Carpathians *B. pales* population, but only a comparably small number of males emerged prior to the females (Fig. 1a), as also observed in the High Tauern National Park in the eastern Alps [39]. Nevertheless, a sufficient number of males was present at the beginning of the females’ emergence so that both sexes can benefit from the known advantages of protandry [28–30]. A quite similar phenology was also observed for *Erebia nivalis* in the eastern Alps [40], where this species inhabits extreme high-altitude habitats. Apparently, conditions in really extreme habitats may be so unpredictable that protandry must be softened to guarantee the survival of butterfly populations in more hostile years and not risking a complete temporal mismatch between both sexes.

This “soft” protandry in the Bucegi *B. pales* population was combined with a month-long phase of mostly constant age structure (Fig. 2), a phenomenon also observed in an eastern Alpine population of the species [39]. This phase of constant age structure was due to an extended emergence period (reflected in the Net Birth rates and the rate of proportional recruitment) resulting in a relatively low average wing damage over time if compared to other butterfly species with mostly simultaneous emergence and hence continuously ageing population structures over the entire observed flight period [34, 37, 54, 86–88]. This capability of constant rejuvenation reduces the species’ susceptibility to short-term bad weather events such as cold snaps [3], one main trigger of increased mortality rates in butterflies [89], which hence reduces the time available for successful reproduction [90, 91]. This form of risk spreading (i.e. bet-hedging [92–94]) against the unpredictable conditions in high mountain environments, combined with the observed “soft” protandry calls for a generally good adaptation of the phenology of the local populations of *B. pales* to the climatic conditions prevailing in the Southern Carpathians.

### Sex-dependent behavioural differences

Comparing the general behavioural and movement traits between sexes, the distances moved by males were significantly larger than for females (Fig. 5 and Table 5), which consequently were observed flying much less, but visited flowers to obtain nectar much more often than males (Tab. 5). Similar behavioural differences between sexes are a common phenomenon in butterflies [35, 37, 41–43].

These differences in flight activity and movement patterns, in combination with the different roles of the sexes (males: finding females; females: successful egg production and oviposition), might also be responsible for the sexual dimorphism in nectar source selection observed in our study, most likely driven by differential nutritional needs. In the Bucegi Mountains (this study) and in the Alps [5], *B. pales* exhibited high specialisation on some few plant species, even down to the species level, but with a strong difference between sexes.

As already known, the selection of nectar sources by butterflies mainly depends on the offered amounts of hexoses, sucrose, fructose and amino-acids, with sugars being more important for flight activity and amino-acids more for egg production [44–46]. Nevertheless, the sex-dependent preferences and differential needs for nectar ingredients continue to be discussed controversially. This specialisation could become problematic in times of climate change, in particular in high mountain regions because it might lead to shifts in the timing of plant flowering and butterfly appearances [95], causing problems for butterflies to find sufficient nectar plants if their phonologies are disengaged from the flowering periods of their preferred nectar sources.

### Mobility and the impact of habitat quality

Habitat quality is influenced by abiotic and biotic factors [96, 97]. Consequently, human activities have paramount importance today. For example, a non-adapted grazing intensity often produces pastures with a low diversity and abundance of flowering plants [98], a situation also observable in our study area in the Bucegi Mountains [26, 27]. Consequently, negative effects of grazing on population sizes and general diversity of invertebrate species have frequently been reported [99–101]. These negative grazing effects are mostly linked to the reduction of essential resources, such as foraging plants or places for oviposition [102]. In our study, the intensively pastured parts of the study area also had a remarkably lower population density of *B. pales* than the grazed sector. This difference is reflecting a higher habitat quality of the ungrazed sector. Nevertheless, this difference might not be solely the result of the different grazing regimes, but other factors like the slightly different altitudes of both sectors and hence some subtle differences in the climatic (and also other environmental) conditions might have additional influence.

However, habitat quality is not only of paramount importance for the persistence of butterfly species in an area [103–105], but also strongly influences the individuals’ behaviour. Thus, movement activities in general increase with a decrease in habitat quality [106, 107]. This also applies to high mountain butterfly species. For example, differences in the movement behaviour in high and low quality habitats were observed for two *Erebia* species in the Hrubý Jeseník Mountains. Individuals of *E. epiphron* tended to be more sedentary in areas with high habitat quality, and also individuals of *E. sudetica* had a stronger dispersal behaviour in habitats of lower quality [48]. Similar results are known for *Parnassius smintheus*, a species which showed restricted movements on large homogenous alpine grasslands without obvious barriers (i.e. optimum habitats), but considerably higher mobility on low quality patches [12, 48, 108, 109]. In this context, the differences between both sectors observed in our study with respect to population density (Fig. 2a and b) and movement behaviour (Fig. 5a and b) of *B. pales* are fully in line with comparable observations made on other mountain butterfly species. Especially the males on the grazed sector showed long movements and consequently a high potential for long-distance dispersal (Table 5) if compared to males of other alpine butterfly species [39].

More specifically, each of the individuals in our study, independently of its sex, had the option of either selecting a sector with lower habitat quality caused by intensive grazing but also lower competition for oviposition places and, most likely, nectar sources, or a sector with higher habitat quality but also higher general competition.

As the movement between both sectors was not clearly favouring one of the two possible directions, we assume that the habitat quality was still adequate for both sexes on both sectors. This was in clear contrast to observations in other butterfly species, such as *Maculinea nausithous* and *M. teleius* [36], that showed a clear migration in the direction of the more favourable habitats. Nevertheless, although both sectors were used by *B. pales*, the negative consequences of intensive grazing were obvious. Under the initially probably similar abiotic and biotic conditions in both sectors, similar population densities could be expected to have occurred on them (but see above). Consequently, the observed differences were most likely caused by the effects of grazing, thus highlighting the strong impact of non-adapted management on the pastures. Except for some small patches of suitable habitats, most parts of the grazed sector were avoided by butterflies, with important consequences on behaviour and dispersal (ES2).

### Conservation implications

Until the end of the 1990s, land-use in the Southern Carpathians was mostly traditional, involving transhumance. The natural and semi-natural grasslands, supported by this type of use, are of great importance for nature and biodiversity conservation [23]. Structural changes in politics, economy and society have led to an intensification of land-use (e.g. by decoupling the sheep per hectare ratio [110]), causing severe conservation problems, especially for natural and semi-natural grasslands [111].

Hence, other studies in the Bucegi Mountains already have shown changes in the plant composition of this area, where a decline in the grass species *Nardus stricta* was observed in an 18-year study. This (formerly dominant) species was increasingly replaced not only by other forage grasses, such as *Agrostis capillaris* and *Poa pratensis*, but also flowering plants like *Trifolium repens* [27]. Furthermore, the intensive sheep pasturing in the Bucegi Mountains has reduced the number of endemic species as well as the biodiversity and abundance of plants and gastropods [26]. Beside these biotic consequences, negative effects on abiotic factors caused by intensive grazing have also been observed, such as increased water pollution and progressive soil erosion [110]. Such negative influences of intensive grazing on the diversity and abundance of insect groups are also known from other studies [100, 112]. This also applies to other high altitude butterfly species, like the south-eastern Alpine endemic *Erebia calcaria,* which is negatively affected by intensive grazing and fragmentation [99].

The land-use changes in the Bucegi Mountains have not only led to local resource scarcity and habitat fragmentation, but most likely have also caused ehavioural changes in the studied population of *B. pales,* evident in the different movement patterns within the grazed and the ungrazed sectors. In general, movement patterns are influenced by diverse factors, like the trade-off between costs and success of a movement, or the combined effects of internal and external information on individuals [113, 114]. As our study was performed at sectors with originally, most likely, similar biotic and abiotic conditions, we assume that also the observed differences result from the differences in grazing intensity. In combination with the previously mentioned studies, we assume that the current level of grazing intensity in the Bucegi Mountains negatively affects (insect) species and biodiversity in general.

To hinder further disappearance of these high mountain species, conservation measures are necessary. One possible instrument is the Carpathian Convention, a treaty to foster the sustainable development and the conservation of the Carpathian region, signed by seven Carpathian states in 2003 [115]. This convention suggested the implementation of traditional land-use in a sustainable manner, with a focus on „the protection of mountain ecosystems, […], the importance of biological diversity, and the specific conditions of mountains as less favoured areas (Carpathian Convention Art. 7, Paragraph 1). Although this article is broadly defined, it is apparent that sustainable development of the Carpathians is not possible without modifications in land-use intensity towards a grazing with a lower intensity. In the context of high mountain ecosystems, this entails a traditional low-intensity land-use, but not abandonment. Another possible instrument is the European Union Law, in particular the Common Agricultural Policy (CAP). This policy serves to support farms and to ensure the ration of agricultural goods (CAP, Art. 39). However, the actual Common Agricultural Policy 2014–2020 is not suitable to support an appropriate and sustainable agriculture as well as to act adequately against the trend of abandonment of farmland in mountain areas. On the one hand, the ecological demands of the system of benefits is not sufficient to achieve an adapted grazing in mountain areas (e.g. Greening and Cross Compliance; Regulation (EU) No 1307/2013). On the other hand, an insufficient amount of money is spent supporting rural development by the European Agricultural Fund for Rural Development (Regulation (EU) No 1305/2013) to prevent rural migration. This is aggravated by the lack of willingness of most EU member states, including Romania, to use the possibility of an obligatory payment for areas with natural constraints (Art. 46 Regulation (EU) No 1307 /2013). This could be an additional source of income for Romanian farmers in high mountain areas. Hence, it would be desirable to strengthen the situation of mountain farming in the next Common Agricultural Policy 2021–2028 to ensure a permeant and sustainable development of the Carpathian meadows. At the same time, further funding resources would help here to realize nature conservation through concrete management actions. In this context and from a European perspective, so-called LIFE projects would be useful supplements to the law principles; unfortunately, no LIFE project is currently in progress to save extensively used Carpathians meadows; http://ec.europa.eu/environment/life/.

Several studies have shown that light grazing even increases the number and abundance of butterfly species, whereas total long-term abandonment with subsequent succession on the long run reduces butterfly diversity [116–119]. A fast and complete implementation of the Carpathian Convention as well as modifications of the Common Agricultural Policy in the next funding period would therefore be of great importance in counteracting the recently observed habitat degradation, fragmentation and diversity loss.

## Conclusion

Specific adaptations for risk-spreading of *B. pales* to cope with the impact of unpredictable weather conditions of the Carpathian Mountains were revealed in this study. As in the Alps [39], we observed an extended emergence of individuals over the observed flight period, dividing the population’s age structure into three phases. In combination with the detected “soft” form of protandry, in which only a small number of males appeared before females, these adaptations enable the species to conquer short bad weather events, because only the part of the whole population being at the wing by that time might be killed. Comparing both sexes, movement differences between males and females were obvious; male individuals flew larger distances and had a higher flight-activity. This might explain the need of different nectar ingredients, which consequently leads to a dimorphism in foraging behaviour: males preferred nectar sources of Asteraceae, females Caprifoliaceae. Although we were not able to study the ungrazed sector as long as the grazed sector, a deterioration of the habitat quality caused by intensive grazing on the grazed sector in comparison to the ungrazed sector was likely. The permanent grazing might have negatively influenced population density, causing significantly larger flight distances of the individuals and different behaviour at both sectors. Comparably negative results of intensive grazing are already known for other insect groups [100, 112]. Hence, the ongoing land-use change and intensification of grazing in the Southern Carpathians, here in particular the Bucegi Mountains [26, 27], are a threat to the native insects in the whole mountain area. To counter this progress, improvements in the actual management and jurisdiction are needed, to protect these insect species from the consequences of overgrazing (e.g. by establishing areas without or just light grazing). In this context, this study about *B. pales* might provide essentials for further research and a better understanding of high alpine butterflies in the Carpathian Mountains.

## Additional files

Additional file 1: **Table S1.** Daily sampling effort for ungrazed and grazed sector (in minutes) calculated with the daily time in field multiplied with number of workers. **Table S2.** Equation, coefficient of determination (R^2^) and adjusted coefficient of determination (R_adj_^2^) for trend lines of the different polynomic function. (DOCX 23 kb)

Additional file 2: Distribution of capture events of *B. pales* in Parcul Natural Bucegi, Romania (2014). (TIFF 4916 kb)

## Abbreviations

AIC_C_: Akaike Information Criterion
Art: Article
CAP: Common Agricultural Policy
ES: Electronic Supplement
EU: European Union
IPF: Inverse power function
m asl: Metres above sea level
MRR: Mark-release-recapture
NEF: Negative exponential function
No: Number
R^2^: Coefficient of determination
R_adj_^2^: Adjusted coefficient of determination
SD: Standard deviation
SE: Standard error
