# Supplementary material for: Dispersal and adaptation strategies of the high mountain butterfly Boloria pales in the Romanian Carpathians
Source: Front Zool. 2019 Jan 17;16:1. doi: 10.1186/s12983-018-0298-1 (PMC6335762; doi:10.1186/s12983-018-0298-1)
Supplement: Supplementary file 1 — Table S1. Daily sampling effort for ungrazed and grazed sector (in minutes) calculated with the daily time in field multiplied with number of workers. Table S2. Equation, coefficient of determination (R2) and adjusted coefficient of determination (Radj2) for trend lines of the different polynomic function. (DOCX 23 kb) [file 12983_2018_298_MOESM1_ESM.docx]

**Table 1: Daily sampling effort for ungrazed and grazed sector (in minutes) calculated with the daily time in field multiplied with number of workers.** Differences in sampling effort base on the greater handling time with the increasing number of butterflies for each day, as well as breaks, later start time or earlier ending because of harsher weather events; these differences were included in the calculations for the Population demography with MARK 8.0 (factor *hours*).

| Date | Number Workers | Time Grazed Sector | Time Ungrazed Sector |
| --- | --- | --- | --- |
| 02.07.2014 | 2 | 300 | - |
| 03.07.2014 | 2 | 360 | - |
| 05.07.2014 | 2 | 480 | - |
| 06.07.2014 | 2 | 530 | - |
| 08.07.2014 | 2 | 510 | - |
| 10.07.2014 | 2 | 630 | - |
| 13.07.2014 | 2 | 380 | - |
| 15.07.2014 | 3 | 815 | - |
| 16.07.2014 | 3 | 690 | - |
| 20.07.2014 | 3 | 825 | - |
| 21.07.2014 | 3 | 870 | - |
| 22.07.2014 | 3 | 560 | - |
| 26.07.2014 | 3 | 575 | - |
| 27.07.2014 | 3 | 820 | - |
| 31.07.2014 | 3 | 885 | - |
| 01.08.2014 | 3 | 855 | - |
| 02.08.2014 | 3 | 455 | - |
| 03.08.2014 | 3 | 825 | - |
| 04.08.2013 | 3 | 405 | - |
| 07.08.2014 | 3 | 540 | - |
| 09.08.2014 | 3 | 690 | - |
| 11.08.2014 | 3 | 195 | - |
| 12.08.2014 | 3 | 720 | - |
| 13.08.2014 | 3 | 750 | 160 |
| 14.08.2014 | 3 | 660 | 160 |
| 15.08.2014 | 3 | 505 | 150 |
| 19.08.2014 | 3 | 540 | - |
| 20.08.2014 | 3 | 670 | 145 |
| 21.08.2014 | 3 | 600 | 210 |
| 25.08.2014 | 4 | 435 | 260 |
| 26.08.2014 | 3 | 240 | 160 |
| 27.08.2014 | 3 | 605 | - |
| 29.08.2014 | 3 | 320 | 205 |
| 30.08.2014 | 3 | 495 | 165 |
| 31.08.2014 | 3 | 420 | 190 |
| 02.09.2014 | 3 | 600 | 195 |

**Table 2: Equation, coefficient of determination (*R²*) and adjusted coefficient of determination (*R_adj_²*) for trend lines of the different polynomic function;** calculated from the age structure calculated from daily wing condition of *B. pales;* the fourth-degree polynomic function obtaining the best adjusted coefficient of determination and coefficient of determination.

| Name | Equation | *R²* | *R_adj_²* |
| --- | --- | --- | --- |
| Second-degree polynomic function | y = 5E-05x² - 4.18x + 88,807 | 0.49 | 0.45 |
| Third-degree polynomic function | y = 1E-05x³ - 1.86x² + 79,085x - 1E+09 | 0.60 | 0.54 |
| Fourth-degree polynomic function | y = 8E-07x^4^ - 0.14x^3^ + 8,725.23x^2^ - 2E+08x + 3E+12 | 0.66 | 0.60 |
| Fifth-degree polynomic function | y = 2E-08x^5^ - 0.004x^4^ + 371.82x^3^ - 2E+07x^2^ + 3E+11x - 3E+15 | 0.67 | 0.59 |
| Sixth-degree polynomic function | y = 8E-10x^6^ - 0.0002x^5^ + 22.47x^4^ - 1E+0.6x^3^ + 4E+10x^2^ - 7E+14x + 5E+18 | 0.67 | 0.58 |
