# Supplementary figures and images for: Dispersal and adaptation strategies of the high mountain butterfly Boloria pales in the Romanian Carpathians
Source: Front Zool. 2019 Jan 17;16:1. doi: 10.1186/s12983-018-0298-1 (PMC6335762; doi:10.1186/s12983-018-0298-1)

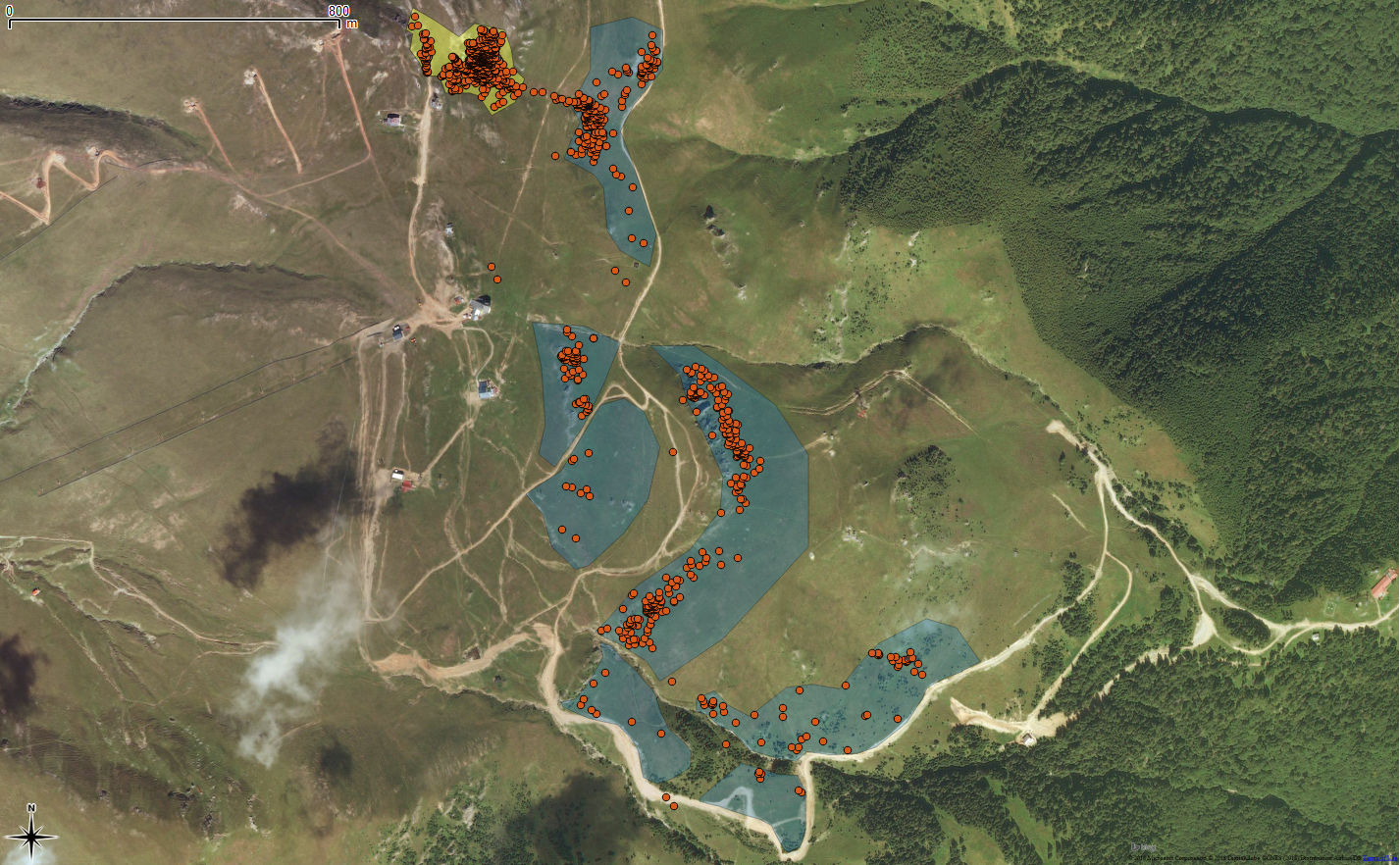

Supplement: Supplementary file 2 — Distribution of capture events of B. pales in Parcul Natural Bucegi, Romania (2014). (TIFF 4916 kb) [file 12983_2018_298_MOESM2_ESM.tiff]
